# Spontaneous Regression of Primary Malignant Lymphoma of the Prostate

**DOI:** 10.1155/2013/363072

**Published:** 2013-09-17

**Authors:** Yoshio Monzen, Mitsuru Nakahara, Takashi Nishisaka

**Affiliations:** ^1^Department of Radiology, Hiroshima Prefectural Hospital, 1-5-54 Ujinakanda Minami-ku, Hiroshima 734-8530, Japan; ^2^Department of Urology, Hiroshima Prefectural Hospital, Japan; ^3^Department of Pathology, Hiroshima Prefectural Hospital, Japan

## Abstract

We herein report a case of primary lymphoma of the prostate, which arose in an 85-year-old male with dysuria. CT and MRI examinations demonstrated a large mass in the prostate. A transrectal ultrasound-guided biopsy of the prostate was performed. The histological examination showed diffuse large B-cell lymphoma. The large lesion in the prostate showed spontaneous regression. Spontaneous regression of primary lymphoma of the prostate has not been reported previously. The spontaneous regression of primary lymphoma of the prostate observed in this patient suggests that observation may represent a viable treatment option following a biopsy that has provided a histopathological diagnosis.

## 1. Introduction

Primary malignant lymphoma of the prostate is rare. We herein report an unusual case of primary lymphoma of the prostate which showed spontaneous regression. Spontaneous regression of primary lymphoma of the prostate has not been previously reported. 

## 2. Case Report

 An 85-year-old male was referred to our hospital due to dysuria in November 2009. The patient had been diagnosed with prostatic hypertrophy in 2003. His serum prostate-specific antigen (PSA) level was 3.4 ng/mL (normal: ~4 ng/mL). Although the patient underwent treatment for prostatic hyperplasia, the dysuria worsened. 

**Figure 1:**
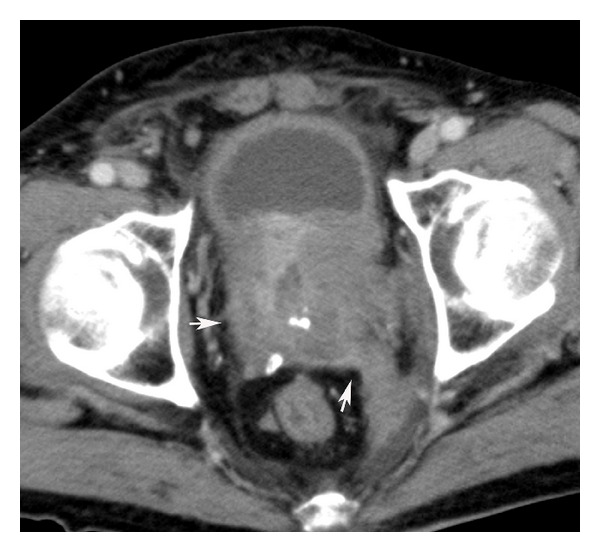
Contrast-enhanced CT demonstrated a large heterogeneous mass (arrow).

 Contrast-enhanced CT demonstrated a large heterogeneous mass involving the prostate, boundary of the bladder, and left ureter (Figure 1). Left hydronephrosis, hydroureter, and two enlarged lymph nodes in the pelvis were also observed. 

**Figure 2:**
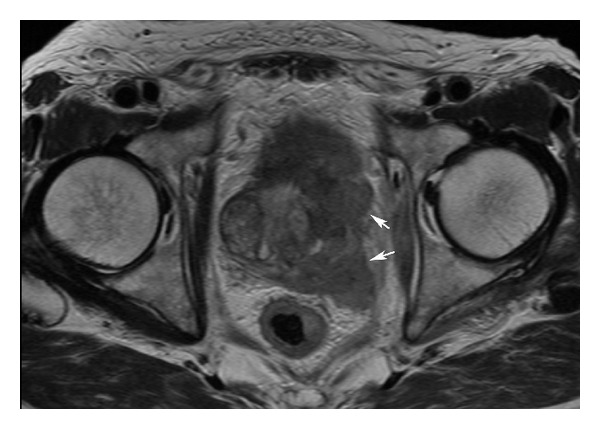
T2-weighted MR imaging demonstrated a tumor originating from the left peripheral zone of the prostate (arrow).

The tumor was found to be hypointense and was shown to have originated from the left peripheral zone of the prostate on T2-weighted MR images (Figure 2). The tumor had infiltrated the seminal vesicles, rectum, and bladder. In summary, the imaging studies revealed a large pelvic mass originating from the prostate. A sarcoma-like tumor was suggested.

**Figure 3:**
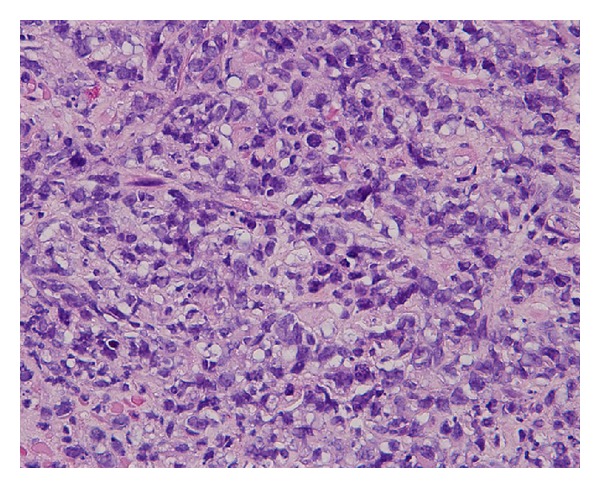
A histological section showing a diffuse large B cell lymphoma (H&E).

 A biopsy of the prostate was performed and identified diffuse large B-cell lymphoma. The histological examination of the hematoxylin and eosin stained sections showed the infiltration of polygonal atypical cells, which were immunohistochemically positive for CD20 and LCA and negative for CD56 (Figure 3). 

Because the patient refused to receive chemotherapy or radiotherapy, the urologists elected to watch the patient closely.

**Figure 4:**
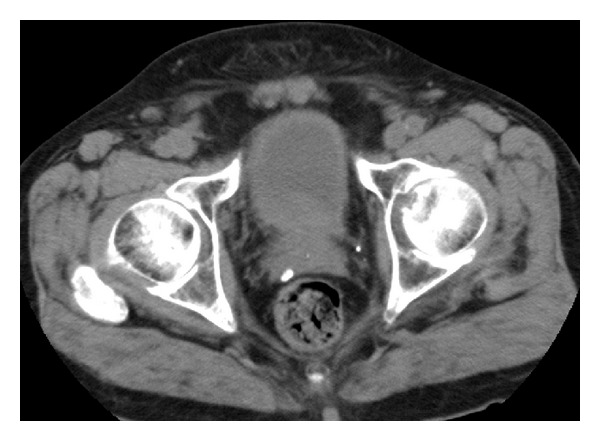
Precontrast CT demonstrated a diminution of the size of the lymphoma of the prostate 25 months after the prostate biopsy.

Precontrast CT demonstrated a diminution of the size of the lymphoma after the prostate biopsy (Figure 4). The two enlarged lymph nodes in the pelvis disappeared, and both the left hydronephrosis and the hydroureter improved.

There has been no local recurrence or distant metastasis in the 31 months since the prostate biopsy. 

## 3. Discussion

 Primary malignant lymphoma of the prostate represents 0.09% of prostate neoplasms and 0.1% of all non-Hodgkin lymphomas [1]. The ages of the reported patients ranged from 5 to 89 years (mean, 62 years). The majority of the cases were of diffuse large B-cell lymphomas, but any type can occur, including Hodgkin's lymphoma, angiotropic lymphoma and MALT lymphoma [2, 3]. 

The criteria used to classify the primary lymphoma of the prostate include that the main symptoms are urinary, the disease occurs predominantly in the prostate with or without extension to adjacent tissues, and there is no involvement of the lymph nodes, liver, spleen, or blood for up to one month after the diagnosis [4]. Our case fulfilled these criteria, therefore and the lymphoma was classified as a primary lymphoma. 

There have been few reports about the imaging of primary lymphoma of the prostate. Claikens et al. reported that there were heterogeneity of the mass on contrast-enhanced CT and on T2-weighted MR images and a moderate increase in signal intensity on contrast-enhanced T1-weighted MRI for non-Hodgkin's lymphoma of the prostate [5]. A hypointense mass originating from the prostate on T2-weighted MR images was found in our patient and was initially considered to be prostate cancer or a sarcoma-like tumor (leiomyosarcoma or rhabdomyosarcoma), and it was difficult to make the differential diagnosis based on the imaging findings, so the biopsy was required to provide the definitive diagnosis.

Several therapeutic modalities have been reported, including prostatectomy, radiotherapy, and chemotherapy. Radical surgery is not indicated, since the local disease can be well controlled with chemotherapy or radiotherapy [1]. Although the prognosis of primary lymphoma of the prostate was classically considered to be poor, some articles have suggested that prolonged survival could be expected if chemotherapy was administered. Taleb et al. reported that combination chemotherapy with rituximab and CHOP was useful for the treatment of diffuse large B cell lymphoma of the prostate [6].

Spontaneous regression of primary lymphoma of the prostate has not been previously reported. However it was reported that a MALT lymphoma of the conjunctiva, lungs, and stomach showed spontaneous regression [7, 8]. However, the mechanism responsible for the spontaneous regression remains unclear. It is thought that the spontaneous regression of lymphoma of the prostate without specific therapy is probably attributable to an immunological response by the host that arises as a result of the injury due to an infection with bacteria or viruses or the injury caused by the biopsy of the prostate [9]. We believe that the etiology of the spontaneous regression of the primary lymphoma of the prostate observed in our present patient was also the result of the biopsy taken for the histopathological diagnosis. Therefore, observation may also be a valid treatment option for lymphoma of the prostate in elderly patients. In support of this hypothesis, a patient reported by Wiernik died 13 years after the spontaneous regression of lymphoma [10]. A careful follow-up for a longer period of time will be necessary in our patient, but he has remained free from recurrence or distant metastasis for 31 months after the biopsy.

## 4. Conclusions

 The spontaneous regression of primary lymphoma of the prostate observed in this patient suggests that, following biopsy for a histopathological diagnosis, observation is a valid treatment option, especially in elderly patients.
